# Hairy cell leukemia and COVID-19 adaptation of treatment guidelines

**DOI:** 10.1038/s41375-021-01257-7

**Published:** 2021-05-04

**Authors:** Michael Grever, Leslie Andritsos, Versha Banerji, Jacqueline C. Barrientos, Seema Bhat, James S. Blachly, Timothy Call, Matthew Cross, Claire Dearden, Judit Demeter, Sasha Dietrich, Brunangelo Falini, Francesco Forconi, Douglas E. Gladstone, Alessandro Gozzetti, Sunil Iyengar, James B. Johnston, Gunnar Juliusson, Eric Kraut, Robert J. Kreitman, Francesco Lauria, Gerard Lozanski, Sameer A. Parikh, Jae Park, Aaron Polliack, Farhad Ravandi, Tadeusz Robak, Kerry A. Rogers, Alan Saven, John F. Seymour, Tamar Tadmor, Martin S. Tallman, Constantine S. Tam, Enrico Tiacci, Xavier Troussard, Clive Zent, Thorsten Zenz, Pier Luigi Zinzani, Bernhard Wörmann

**Affiliations:** 1 Division of Hematology, Department of Medicine, The Ohio State University, Columbus, OH USA; 2 Division of Hematology Oncology, University of New Mexico Comprehensive Cancer Center, Albuquerque, NM USA; 3 Department of Internal Medicine & Biochemistry and Medical Genetics, Rady Faculty of Health Sciences, University of Manitoba, Winnipeg, MB Canada; 4 Research Institute in Oncology and Hematology, CancerCare Manitoba, Winnipeg, MB Canada; 5 Department of Medicine, Northwell Health Cancer Institute, Donald and Barbara Zucker School of Medicine at Hofstra/Northwell and Feinstein Institute for Medical Research, New York, NY USA; 6 Division of Hematology, Mayo Clinic, Rochester, MN USA; 7 Royal Marsden Hospital, London, UK; 8 Department of Internal Medicine and Oncology, Semmelweis University, Budapest, Hungary; 9 Department of Hematology, University Hospital, Heidelberg, Germany; 10 Institute of Hematology, Department of Medicine and Surgery, University and Hospital of Perugia, Perugia, Italy; 11 Cancer Sciences and Haematology Department, University of Southampton Hospital Trust, Southampton, UK; 12 Johns Hopkins University, Baltimore, MD USA; 13 University of Siena Policlinico S Maria alle Scotte, Siena, Italy; 14 Department of Internal Medicine, University of Manitoba, Winnipeg, MB Canada; 15 Stem Cell Centre, Department of Laboratory Medicine, Lund University, and Department of Hematology, Skåne University Hospital, Lund, Sweden; 16 Laboratory of Molecular Biology, National Cancer Institute, NIH, Bethesda, MD USA; 17 Department of Pathology, The Ohio State University Medical Center, Columbus, OH USA; 18 Department of Medicine, Memorial Sloan Kettering Cancer Center, New York, NY USA; 19 Hadassah—Hebrew University Medical Center, Jerusalem, Israel; 20 Department of Leukemia, University of Texas MD Anderson Cancer Center, Houston, TX USA; 21 Department of Hematology, Medical University of Lodz, Lodz, Poland; 22 Division of Hematology and Oncology, Scripps Clinic, La Jolla, CA USA; 23 Haematology Department, Peter MacCallum Cancer Centre and Royal Melbourne Hospital, University of Melbourne, Melbourne, VIC Australia; 24 Hematology Unit, Bnai-Zion Medical Center and Rappaport Faculty of Medicine, Technion Institute Technology, Haifa, Israel; 25 Department of Hematology, Centre Hospitalier Universitaire Côte de Nacre, Caen, France; 26 James P. Wilmot Cancer Institute, University of Rochester Medical Center, Rochester, NY USA; 27 Department of Medical Oncology and Haematology, University Hospital Zürich and University of Zürich, Zürich, Switzerland; 28 IRCCS Azienda Ospedaliero-Universitaria di Bologna and Instituto di Ematologia “Seràgnoli”, Dipartimento di Medicina Specialistica, Diagnostica e Sperimentale Università degli Studi, Bologna, Italy; 29 Charité-University Medicine, Berlin, Germany

**Keywords:** Diseases, Leukaemia

## Abstract

Standard treatment options in classic HCL (cHCL) result in high response rates and near normal life expectancy. However, the disease itself and the recommended standard treatment are associated with profound and prolonged immunosuppression, increasing susceptibility to infections and the risk for a severe course of COVID-19. The Hairy Cell Leukemia Foundation (HCLF) has recently convened experts and discussed different clinical strategies for the management of these patients. The new recommendations adapt the 2017 consensus for the diagnosis and management with cHCL to the current COVID-19 pandemic. They underline the option of active surveillance in patients with low but stable blood counts, consider the use of targeted and non-immunosuppressive agents as first-line treatment for cHCL, and give recommendations on preventive measures against COVID-19.

## Introduction

Severe acute respiratory syndrome coronavirus 2 (SARS-CoV-2) is the cause of coronavirus disease 2019 (COVID-19) and continues to have a major impact on global health care and clinical practice. Cancer patients, particularly those with hematological malignancies, seem to be at higher risk for a severe course of the infection [1–12]. While there are encouraging developments for broad access to effective and safe vaccines as well as therapeutic agents, the recent global increases in case numbers coupled with identification of novel strains with increased transmission rates emphasize the need to understand the outcome in hairy cell leukemia (HCL) and consider our management of patients with this rare hematologic malignancy.

HCL is an uncommon hematological malignancy. Modern treatment options in classic HCL (cHCL) result in high response rates and near normal life expectancy [13, 14]. However, the disease itself and the recommended standard treatment are associated with profound and prolonged immunosuppression and increased susceptibility to severe infections [15]. A recent review of the clinical course of patients with chronic lymphocytic leukemia (CLL) who have COVID-19 shows the high mortality associated with the need for hospitalization, increased patient age, and the number of co-morbid conditions [12, 16]. At present, there are no updated published data on the infection rates with SARS-CoV-2 in HCL patients. Experiences with the course of COVID-19 in HCL patients are limited to case reports [17, 18], and personal communications. Despite this lack of evidence, HCL patients and their treating physicians ask for guidance.

## Methods

The Hairy Cell Leukemia Foundation (HCLF) has recently convened experts and discussed different clinical strategies for the management of these patients. Recommendations are based on the 2017 consensus for the diagnosis and management with cHCL, and on the increasing evidence on the adverse course of COVID-19 in patients with hematological malignancies [13]. Consequently, some recommendations of the published guidelines require adaptation.

Considering that most patients with cHCL present with varying degrees of pancytopenia, these individuals may have a concomitant ongoing infection that requires immediate treatment. Before embarking upon standard therapy with a purine analog, which is both myelosuppressive and immunosuppressive, it is important to control the active infection. The dilemma of adequately treating infection in the patient with pancytopenia requires careful consideration. In a large Italian study reviewing the clinical findings in this disease, 39% of patients had an absolute neutrophil count <0.5 × 10^9^/L [19]. It had been estimated that 17% of newly diagnosed cHCL patients have an active infection at presentation [20]. Consequently, selecting the appropriate agents and timing for therapy requires considerable thought. In patients with an absolute neutrophil count <1.0 × 10^9^/L, consideration is usually given to initiate therapy before the counts further decline to even lower levels or the patient actually develops an infection. In patients with mild neutropenia during this COVID-19 pandemic who are not actively infected, they may temporarily delay therapy for the leukemia for a period of time if they are followed very closely to avoid missing a significant decline in blood counts that would place them at risk for serious infection. This approach may be limited to those patients with mild reduction in hematologic parameters, and thus avoid immunosuppressive therapy in the midst of the COVID-19 surge. This approach may also enable successful immunization before the exposure to definitive therapy for the leukemia.

In addition to severe neutropenia, patients with cHCL have profound monocytopenia and abnormalities in other immune effector cells. In addition, the standard therapeutic agents associated with a high complete remission rates in this disease, cladribine and pentostatin, produce profound and prolonged immunosuppression [21, 22]. Following therapy with these agents, cHCL patients frequently experience severe lymphocytopenia that may require a year or more to recover. Another important agent often utilized in combination with purine analogs involves an anti-CD20 monoclonal antibody (e.g., rituximab or obinutuzumab), which can also increase the risk of infection either by reducing normal B cells or absolute granulocytes.

Consequently, patients in need of effective therapy for their leukemia are at increased risk for bacterial, viral, and opportunistic infection. The serious nature of these infections results both from the intrinsic immunosuppression associated with this leukemia, and its treatment. Several reports have highlighted the increased risk of poor outcome following COVID-19 infection in patients with a hematologic malignancy, but the comparative experience in cHCL has not been specifically well-documented [3, 10, 23, 24]. Since late in 2019, the progressively increasing threat of COVID-19 infection across the globe has led hematologists to consider the impact of using these highly effective, yet immunosuppressive agents on the outcome for patients who need antileukemic therapy. However, there is currently a paucity of data regarding the impact of treatment on the outcome of COVID-19 in these immunocompromised patients. While we collect adequate clinical experience to issue data-driven recommendations, the HCLF sponsored a panel discussion of experts to provide interim recommendations considering the serious consequences for patients acquiring this infection. At present, our best efforts have been directed to preventing exposure to COVID-19 while the development of effective strategies for immunization is being vigorously pursued.

### Recommendations

#### Prevention

Cancer patients are strongly recommended to follow the national and local guidelines for prevention of the further spread of SARS-CoV-2. These include social distancing, hand hygiene, and surgical masks covering nose and mouths (https://www.hematology.org/covid-19) (https://ehaweb.org/topics-in-focus/covid-19/covid-19-recommendations/) [25]. Several highly effective vaccines have been recently developed with specific focus on decreasing the risks of acquiring this virus, and vigorous efforts at immunization are underway. The two currently available in the United States and Europe, mRNA-based vaccines have a protection rate of >90%. Severe adverse events are rare. Duration of protection is unknown. In analogy to experiences with influenza, there is concern of reduced efficacy of anti-SARS-CoV-2 vaccines in patients under or immediately after treatment with anti-CD20 antibodies [26–29]. Vaccination in HCL patients may be delayed to up to 6 months or longer after the last application of anti-CD20 antibodies. Considering the T-cell-mediated action of the mRNA-based vaccines, the concern of reduced efficacy may also apply for patients under or after treatment with purine analogs [30]. Thus far, there is no indication for a negative effect of the currently approved anti-Sars-CoV-2 vaccines in the individual, immunocompromised patient. Patients with hairy HCL should be encouraged to receive vaccination against anti-SARS-CoV-2 unless specific contraindications exist despite the uncertainty of response. Confirmation of a serologic response following vaccination would be helpful.

In addition to speedy access to vaccines for SARS-CoV-2 thought to be effective and safe, an effort should also be made to assure that other immunizations are up to date in these immunocompromised patients (e.g., annual influenza vaccination and pneumococcal vaccinations).

HCL patients should use all available options to communicate with their hematologists without physical contact and to reduce potential exposure to SARS-CoV-2 in the health care environment. The marked increase in the use of telemedicine has enabled close patient monitoring in those patient communities with resources and adequate internet connection. Follow-up intervals may be prolonged in asymptomatic patients with stable hematological remission. Adequate access to testing for COVID-19 must be readily available. Patients must have access to the medical team if infection arises or side effects from therapy are encountered.

HCL patients diagnosed with SARS-CoV-2 infection should undergo either self-quarantine, single room, or cohort isolation. While infectiousness of SARS-CoV-2 generally declines within 7–8 days after onset of symptoms, prolonged shedding of viral RNA has been observed in immunocompromised patients indicating that the quarantine for immunocompromised patients with HCL should be extended to at least 20 or more days following the onset of symptoms of viral infection [31–33]. SARS-CoV-2 PCR testing should be available to all HCL patients at increased risk for infection and for monitoring after documented exposure. Documentation of the presence of IgG antibodies in patients who have recovered will be useful in understanding the response to this infection. In patients with minimal to no symptoms who are recently diagnosed, multiple therapeutic strategies are under exploration e.g., antiviral drugs and monoclonal antibodies to reduce the viral load, anti-IL-6 antibodies, and BTK inhibitors as anti-inflammatory agents, convalescent plasma to enhance the immune response, and others.

#### Newly diagnosed cHCL—start of treatment

The 2017 consensus statement described criteria for treatment of cHCL [13]. These guidelines were developed in an era before the onslaught of the SARS-CoV-2 pandemic. They are mainly based on the decline of hematological parameters, but also consider clinical symptoms and the individual course of the disease. Since active cancer including advanced and progressing hematological malignancies are associated with increased mortality in patients with COVID-19 [2–12], start of antineoplastic treatment should not be unnecessarily delayed in HCL patients. The fear of a potential complication, e. g., with SARS-CoV-2, should not jeopardize the effective treatment of an untreated existing life-threatening disease like HCL.

Considering the consequences of untreated progressive pancytopenia, initiation of effective therapy should not be delayed in the absence of ongoing infection. Since many patients with newly diagnosed cHCL have pancytopenia, it may be prudent to initiate therapy before the hematologic parameters decline to critical levels. In a small number of patients, however, who are asymptomatic at diagnosis with only mild reduction in blood counts, they may be followed closely and therapy started when the blood counts show evidence of early decline. While the Consensus Guidelines provide discrete values for initiating antileukemia therapy, it is important to use clinical judgment coupled with close follow-up to determine whether a specific patient should be started on therapy during this pandemic. Furthermore, there is an opportunity to select specific therapeutic agents in conjunction with patient input that may minimize the immediate risk of serious immune suppression during this highly dangerous period of COVID-19 pandemic.

In the patient with pancytopenia and an active uncontrolled infection, treatment with a purine analog-based regimen may be associated with prolonged granulocytopenia. Several less myelosuppressive and immunosuppressive regimens have been reported to effectively improve the hematologic parameters enabling control of infection in conjunction with appropriate antibiotics, antifungal or antiviral agents. The “off-label” use of a BRAF inhibitor (e.g., vemurafenib) in cHCL patients harboring this mutation has resulted in disease responses including early granulocyte recovery enabling control of infection [34]. Subsequent to stabilization of the patient with control of infection, additional therapy may be considered to secure adequate disease control of the leukemia. BRAF inhibitors alone result in both complete and partial responses in BRAF V600E mutated cHCL. The duration of response is longer in patients with a complete response, but shorter in those who achieve a partial response [35].

The combination of a BRAF inhibitor either with a monoclonal anti-CD20 antibody or a MEK inhibitor can further increase the rate of durable remissions without inducing severe myelosuppression [36, 37]. Therefore, selection of the optimal induction therapy for the newly diagnosed patient should consider these “off-label” options that may avoid excessive myelosuppression during the current pandemic. It may also be prudent to avoid the use of an anti-CD20 monoclonal antibody during this time as well if the patient is scheduled to be soon vaccinated against SARS-CoV-2 (see section on prevention) or if the benefit of adding rituximab in terms of depth and rapidity of response is offset by the perceived risk of rituximab-induced immune suppression.

#### Newly diagnosed cHCL—choice of treatment

Primary treatment over the past two decades includes either cladribine or pentostatin [13]. Both nucleoside analogs are effective and may induce long-term complete remissions. Purine nucleosides have been the established approved therapy for cHCL either as front-line therapy or for those in relapse. Their use has resulted in marked improvement in survival in patients with HCL, but both agents are very immunosuppressive. Different schedules have been proposed for cladribine. The administration on 5 consecutive days of cladribine is frequently used as highly effective induction, but is myelosuppressive. Other schedules have been explored in an effort to reduce myelosuppression, but have been equivalent with respect to infectious complications [38, 39]. Pentostatin can also be myelosuppressive, but the schedule of administration enables dose titration to reduce the depth and length of myelosuppression [40]. Both cladribine and pentostatin cause serious and prolonged immunosuppression and their use requires careful consideration during the COVID-19 pandemic.

The combination of cladribine with rituximab decreases the MRD positivity rate versus cladribine monotherapy in first-line therapy [41]. Rituximab, however, has an additional immunosuppressive effect. While the combination of cladribine and rituximab is often used for cHCL patients in relapse, there has been a trend to use them as initial induction in an effort to reduce the extent of residual disease. Considering the potential contribution of rituximab to further impairment in immunity and its ability to decrease the effectiveness of subsequent immunization for many months, some hematologists are re-considering the risk–benefit assessment of using this combination of a purine analog and a monoclonal antibody at this time in the midst of a pandemic. An additional challenge is the treatment of cHCL patients with active infection and a high risk of SARS-CoV-2 infection. In these patients with the current extent of the pandemic, alternatives to nucleoside analogs are being considered:

– BRAF inhibitors

- Vemurafenib induces rapid responses without myelosuppression [34, 35, 42]. Responses are expected to be of shorter duration than with nucleoside analogs. Thus, treatment with BRAF inhibitors may be considered as bridging to a later treatment with one of these chemotherapeutic agents. However, additional therapy with other agents should be added when cytopenias develop indicating relapse after stopping the BRAF inhibitor. Other BRAF inhibitors (dabrafenib, encorafenib) are also expected to be effective, but the database is smaller.
- The duration of response to vemurafenib relapsed or refractory cHCL was related to the quality of remission achieved. Patients achieving a complete remission had a median remission duration of 19 months after stopping treatment, while those with a partial remission had shorter durations of disease control (median of 6 months) [35].
- As monotherapy delivered over 2–6 months in prospective clinical trials [35], the standard dose of vemurafenib (960 mg twice daily) was often reduced for toxicity. Consequently, based on a retrospective case series of 21 patients showing antileukemic activity of lower drug doses [42], some hematologists in practice have utilized lower doses with the intention of avoiding the side effects. The actual optimal dose for a BRAF inhibitor in this disease has never been fully defined. The doses that had been selected for the initial trials in HCL were similar to those used in treating patients with metastatic melanoma.
- Tiacci and colleagues in a prospective clinical trial on 31 cHCL patients have recently shown that the combination of vemurafenib (delivered for just 8 weeks) with an anti-CD20 monoclonal antibody (rituximab) can achieve complete remissions in >90% of patients with relapsed or refractory disease with acceptable toxicity. Hematologic recovery is rapid, and associated with a relatively short course of combined therapy [43].
- The use of a BRAF inhibitor as a “bridge” therapy for patients with active infection during this pandemic is therefore also worthy of consideration. Most experience has been obtained with vemurafenib; however, dabrafenib monotherapy [44] and the combination of dabrafenib with trametinib as an effective strategy for BRAF and MEK inhibition have also been reported to be effective in securing control of the leukemia without prolonged myelosuppression [37]. It should be emphasized that the use of these novel targeted agents has been exciting, but they are not officially approved for the treatment of HCL. While these agents are approved by the FDA and other agencies for the treatment of other malignant diseases, their use in cHCL is “off-label.” Consequently, more investigation will be required before their use will be readily available. These agents can produce highly effective responses with less myelosuppression and immunosuppression than standard therapy, and consequently are of potential value in treating patients in danger of infection. However, additional long-term investigation is required to determine their optimal doses and schedules of administration. Their potential use as “bridge therapy” during the pandemic is promising. Further investigation of the impact of these new targeted agents on the treatment of the disease compared to purine analogs will be needed in the future.

– Interferon (IFN) alpha

- IFN alpha also induces remissions in cHCL patients without the neutrophil nadir associated with nucleoside analogs [45]. Time to remission may take up to several months. Most recently, IFN alpha preparations have been taken off the market by major producers due to declining sales, but pegylated formulations are still available. While IFN has been used for a long time in cHCL, its use was felt to be less effective or durable than the purine analogs. However, this agent may have definite benefit for patients with infection complicated by pancytopenia in this era of the pandemic.

#### Relapsed cHCL

Most cHCL patients are followed at regular intervals. Relapse becomes evident months or even years before another treatment is required. While treatment of patients in relapse was often approached with a plan to use a combination of a purine analog and an anti-CD20 monoclonal antibody, concerns regarding excessive immunosuppression during this pandemic have resulted in alternative strategies. Asymptomatic patients with only mild reduction in blood counts may be followed closely until blood counts show evidence of further decline, especially if vaccination against SARS-CoV-2 is already scheduled in a short time. If treatment is indicated, several agents may be considered including vemurafenib in patients who harbor the BRAF mutation. BRAF inhibitors vemurafenib alone or with rituximab induce rapid responses without myelosuppression and with no or less immune suppression compared to chemo-immunotherapy [35, 42]. The other BRAF and MEK inhibitors may also be considered [37, 44].

Preliminary studies with a BTK inhibitor (Ibrutinib) have been reported to induce responses that are associated with a prolonged period of PFS [46]. However, these small molecules may also reduce response to vaccines, supporting vaccination at least 3 weeks prior to start of Ibutinib [47, 48].

In 2018, moxetumomab pasudotox, an anti-CD22 recombinant immunotoxin, was approved by the FDA for patients with relapsed or refractory HCL after at least two prior lines of therapy. Efficacy is high with an overall response rate of 75% and a complete remission rate of 41% with 30% classified as durable. Side effects include lymphocytopenia, but are generally short [49, 50]. Moxetumomab pasudotox spares T cells and, due to its short half-life, only transiently decreases normal B cells. In some countries, this immunotoxin is available via an expanded access program.

## Discussion

Patients with active cHCL are immunosuppressed and potentially at risk for a severe course of COVID-19. There is a paucity of data describing the impact of this pandemic on the clinical course of this disease. Consequently, the clinical experiences with HCL derived from international centers of excellence should be studied and reported. There are published reports of the impact of COVID-19 in patients with CLL that have some utility for management of cHCL because these distinct B-cell lymphoid malignancies are both immunosuppressive and have overlapping effective therapies. However, it is important to appreciate that while most patients with newly diagnosed CLL do not require immediate therapy, many newly diagnosed patients with cHCL present with severe pancytopenia, and unnecessary delays in initiating treatment should be avoided. However, clinical judgment is required for the asymptomatic patient presenting with mild cytopenia. The plan to “watch and wait” before initiating therapy requires a commitment to close follow-up.

Decisions related to treatment of cHCL or CLL in patients with active infection present a considerable challenge. Fortunately, both lymphoid malignancies can be effectively treated with targeted therapy. In the current environment of a dangerous pandemic that would pose risks to the immunocompromised patients, it is imperative to consider the selection and possible modification in choice of therapy. The currently available drugs enable individualized treatment decisions depending on the patient’s disease profile and the locoregional situation of the COVID-19 pandemic. While treatment for cHCL is fortunate to have several active targeted agents and effective regimens, the much rarer variant of HCL (vHCL) deserves more effort to define effective therapies that are less immunosuppressive. In the vHCL, the absence of the specific BRAF V600E mutation eliminates the possibility for utilizing available BRAF inhibitors. Sequencing the patient’s leukemic cells may identify genomic targets that afford the possibility for using specific agents (e.g., MEK inhibitor) [51]. Therefore, substantial efforts will be needed to identify effective therapy for vHCL that hopefully will spare the patient’s immune system.

All available data on the impact of SARS-CoV-2 on HCL patients should be incorporated into the current registry projects. Utilization of this clinical data will facilitate evidence-based recommendations in selecting the most effective agents with the least myelosuppressive and immunosuppressive consequences while striving for the best response. Effective therapy for these pancytopenic cHCL patients should not be delayed during this pandemic, but consideration for side effects of the therapy must be incorporated into the effort to achieve a solid remission. The course of the SARS-CoV-2 pandemic will hopefully be impacted by the immunization of the community with prioritization for health care workers, first responders, the elderly and the immunocompromised. In addition to the enormous task of effective global immunization, scientific endeavors to develop new therapeutic antiviral agents and close monitoring of the virus for evolution of virulent strains will be necessary. Since HCL is a rare hematologic malignancy with a global distribution, recommendations for management during this pandemic require some adaptations of the published guidelines [13]. As more experience is gained treating HCL during this pandemic, future recommendations for therapy may be based upon data rather than expert opinion.

## Supplementary information

Author Information Excel Spreadsheet

Authors Conflict of Interest and Financial Disclosures
