# Supplementary material for: Hairy cell leukemia and COVID-19 adaptation of treatment guidelines
Source: Leukemia. 2021 May 4;35(7):1864–72. doi: 10.1038/s41375-021-01257-7 (PMC8093591; doi:10.1038/s41375-021-01257-7)
Supplement: Supplementary file 2 — Authors Conflict of Interest and Financial Disclosures [file 41375_2021_1257_MOESM2_ESM.docx]

**COI/Financial Disclosure for Manuscript Authors**

Leslie Andritsos: Previously provided consultation services for Innate Pharma and AstraZeneca

Versha Banerji: VB serves on the advisory boards of Gilead, Lundbeck, Janssen, Astra Zeneca and Abbvie and has had research funding from CIHR, LLSC, CCMF, Roche, Janssen, Abbvie and Lundbeck. VB also received fees from BIOGEN for patented compounds unrelated to this study.

Jacqueline C. Barrientos: Research funding: AstraZeneca, Oncternal, TG therapeutics, Pharmacyclics/AbbVie; Advisory Board: AstraZeneca, Pharmacyclics/Abbvie, Beigene, Genentech, Gilead, Innate

Seema Bhat: Served on Advisory board for Pharmacyclics and Janssen, Beigene and AstraZeneca; Received honorarium from OncLive; Received travel grant from Arqule

James S. Blachly: Consulting and Advisory Board: AbbVie, AstraZeneca, INNATE Pharma, KITE Pharma; Research funding: MingSight Pharmaceuticals: Patents and Intellectual Property: A leukemia diagnostic device (patent pending)

Timothy Call: None

Matthew Cross: None. Research work is funded by the Royal Marden Cancer Charity

Claire Dearden: Has been an advisor for Medimmune/ Innate Pharma (moxetumomab); Consulting/ Advisory board for Abbie and Jansen

Judit Demeter: Has participated in advisory committees in Hungary for Novartis, Bristol Myers Squibb, Amicus, Angelini, Pfizer, Amgen, Roche

Sascha Dietrich: None

Brunangelo Falini: None

Francesco Forconi: None

Douglas E. Gladstone: None

Alessandro Gozzetti: Advisory board: Amgen, Takeda. Research funding: Jansenn and Cilag SPA. Honoraria: Abbvie, Jansenn Cilag, Celgene, Amgen, Takeda

Michael Grever: Consultant: Astra Zeneca, Pharmacyclics, Ascerta, Axio, Inc;

Research Funding: Hairy Cell Leukemia Foundation for Patient Data Registry; Travel Expenses: Hairy Cell Leukemia Foundation; Scientific Board Chair: Hairy Cell Leukemia Foundation Scientific Board (no reimbursement); Scientific Honorarium: University of Pittsburgh

Sunil Iyengar: Advisory and speaker fees: Gilead and Takeda; Advisory: Beigene; Speaker fees: Janssen, Takeda and Gilead

James B. Johnston: None

Gunnar Juliusson: None

Eric Kraut: None

Robert J. Kreitman: Employment: National Institutes of Health and Regional Cancer Care Associates; Honoraria: PlatformQ, OncLive, Cure; Research Funding: Innate, AstraZeneca, Novartis, Genetech, Pfizer, Teva, Hairy Cell Leukemia Foundation; Patents: Coinventor for NIH patent for Moxetumomab Pasudotox

Francesco Lauria: None

Gerard Lozanski: None

Sameer A. Parikh: Research funding has been provided to the institution from Pharmacyclics, Janssen, AstraZeneca, TG Therapeutics, Merck, AbbVie, and Ascentage Pharma for clinical studies in which Sameer A. Parikh is a principal investigator. Sameer A. Parikh has also participated in Advisory Board meetings of Pharmacyclics, AstraZeneca, Genentech, GlaxoSmithKline, Innate Pharma, Adaptive Biotechnologies, and AbbVie (he was not personally compensated for his participation).

Jae Park: Research funding from Genentech, Servier, Takeda, Fate Therapeutics and Amgen. Consulting fees from Servier, Amgen, AstraZeneca, Innate Pharma, Novartis, Kite Pharma, Takeda, Intellia, and Kura Oncology

Aaron Polliack: None

Farhad Ravandi: Consultancy and Honoraria – Celgene, BMS, Amgen, Astellas, Xencor, Agios, AstraZeneca, Orsenix, Innate Pharma, Syros, Taiho, Novartis; Research Funding – BMS, Amgen, Xencor, Macrogenics, Orsenix, Abbvie, Taiho, Prelude, Astex

Tadeusz Robak: Research funding: AstraZeneca, Medimmune, Roche, Janssen, Abbvie, Advisory board; AstraZeneca, Jassen, Abbvie; Travel grant: Roche, Janssen; Honoraria: Abbvie, AstraZeneca, Janssen

Kerry A. Rogers: Received research funding from Genentech, AbbVie, Janssen, and Novartis all paid to her institution, consulted for Acerta Pharma, AstraZeneca, Innate Pharma, Genentech, AbbVie, and Pharmacyclics, received travel funding from AstraZeneca

Alan Saven: Consultant and Advisory Boards: AstraZeneca and Innate Pharmaceuticals; Speakers Bureau: AbbVie and Pharmacyclics

John F. Seymour: AbbVie, Advisory board, speakers’ bureau, research funding; Astra Zeneca, Advisory board; Celgene, Advisory board, speakers’ bureau, research funding, expert testimony; Genentech, Advisory board; Gilead, Advisory Board; Janssen, Advisory board, research funding; Mei Pharma, Advisory board; Morphosys, Advisory board; Roche, Advisory board, speakers’ bureau, research funding, expert testimony; Sunesis, Advisory board; Takeda, Advisory board

Tamar Tadmor: Advisor: Roche, AbbVie, Janssen, Takeda, Gilead, AstraZeneca, Novartis

Martin S. Tallman: Research funding: AbbVie, Amgen, Biosight, Glycomimetics, Orsenix,

Rafael; Advisory boards: Amgen, Daiichi-Sankyo, Delta Fly Pharma, Innate pharmaceuticals, Jazz, Kahr, Kura, Novartis, Orsenix, Roche, Syros; Royalties: UpToDate

Constantine S. Tam: Honoraria from Janssen, AbbVie and Beigene, and his hospital receives research funding from Janssen, AbbVie and Beigene

Enrico Tiacci: Consultant for Innate Pharma. Research funding: Roche. Travel cost: Shire. Holder of a patent on the use of mutant BRAF as HCL biomarker. Our research work in HCL is funded by the Hairy Cell Leukemia Foundation, the Leukemia and Lymphoma Society and the Associazione Italiana Ricerca sul Cancro (AIRC).

Xavier Troussard: Consultant for Innate Pharma, AstraZeneca; Advisor: Abbvie

Bernhard Wörmann: None

Clive Zent: Funding through the University of Rochester for laboratory research, from Acerta/AstraZeneca and TG Therapeutics, and from the Hairy Cell Leukemia Foundation

Thorsten Zenz: Advisor: Roche, AbbVie, Janssen, Takeda, Gilead, AstraZeneca

Pier Luigi Zinzani: Speakers Bureau: Verastem, Celltrion, Gilead, Janssen-Cilag, BMS, Servier, MSD, TG Therap., Takeda, Roche, Eusapharma, Kyowa Kirin; Advisory Board: Verastem, Celltrion, Gilead, Janssen-Cilag, BMS, Servier, Sandoz, MSD, TG Therap., Takeda, Roche, Eusapharma, Kyowa Kirin, ADC Therap.; Consultant: Verastem, MSD, Eusapharma, Sanofi
